# Bacteriological Diagnosis: Tabular Aids for Use in Practical Work

**Published:** 1892-08

**Authors:** 


Bacteriological Diagnosis: Tabular Aids for Use in Practical Work. By James Eisenberg, Ph. D., M. D., Vienna. Translated and augmented with the permission of the author from the second German edition, by Norval H. Pierce, M. D., Surgeon to the Out-Door Department of the Michael Reese Hospital; Assistant to Surgical Clinic, College of Physicians and Surgeons, Chicago, Ill. Philadelphia and London: The F. A. Davis Co., Publishers. 1892.

These tables are arranged in excellent order, and for the laboratory student are exactly what is wanted. The bacteria are classified into pathogenic, non-pathogenic, and fungi. The non-pathogenic are further divided into those which liquefy gelatin, and those which do not liquefy gelatin. The pathogenic are divided into those which can be cultivated outside the human body and those which cannot be so cultivated. Each bacillus or germ is allotted one full page, and here is then tabulated its characteristics, as place found, form and arrangement, mobility, growth, culture, spore formation, aerobiosis, gas production, gelatin reaction, color production, and pathogenesis. Besides, the name and date of the discoverer is given, and the literature where it can be found. An appendix, describing carefully the technique used in the cultivation and staining of bacteria, follows.

Taken as a whole, the tables are of great assistance to students, and the efforts of author and translator are to be commended, while the press-work, by the F. A. Davis Co., is nicely executed.

